# Machine Learning Strategy for Accelerated Design of Polymer Dielectrics

**DOI:** 10.1038/srep20952

**Published:** 2016-02-15

**Authors:** Arun Mannodi-Kanakkithodi, Ghanshyam Pilania, Tran Doan Huan, Turab Lookman, Rampi Ramprasad

**Affiliations:** 1 Department of Materials Science and Engineering, Institute of Materials Science, University of Connecticut, 97 North Eagleville Road, Storrs, Connecticut 06269, USA; 2 Materials Science and Technology Division, Los Alamos National Laboratory, Los Alamos, New Mexico 87545, USA; 3 Theoretical Division, Los Alamos National Laboratory, Los Alamos, New Mexico 87545, USA

## Abstract

The ability to efficiently design new and advanced dielectric polymers is hampered by the lack of sufficient, reliable data on wide polymer chemical spaces, and the difficulty of generating such data given time and computational/experimental constraints. Here, we address the issue of accelerating polymer dielectrics design by extracting learning models from data generated by accurate state-of-the-art first principles computations for polymers occupying an important part of the chemical subspace. The polymers are ‘fingerprinted’ as simple, easily attainable numerical representations, which are mapped to the properties of interest using a machine learning algorithm to develop an on-demand property prediction model. Further, a genetic algorithm is utilised to optimise polymer constituent blocks in an evolutionary manner, thus directly leading to the design of polymers with given target properties. While this philosophy of learning to make instant predictions and design is demonstrated here for the example of polymer dielectrics, it is equally applicable to other classes of materials as well.

The materials design process requires the identification of materials that meet a desired application or property need. The traditional routes adopted thus far to meet such design goals involve the determination of the relevant properties of a large number of potential candidates, via high-throughput experiments or computations, and choosing the best cases for further studies and optimisation^1^,^2^,^3^,^4^. While powerful and successful, this strategy suffers from two primary drawbacks. First, the consideration of each material in a case-by-case manner is laborious and time-consuming, especially if one were to ignore the availability of past data on the same or similar candidate materials. Second, the prevalent strategy addresses the materials design problem in an ‘inverted’ manner, i.e., instead of approaching the “desired properties → suitable materials” design problem (previously referred to as *inverse* design^5^,^6^,^7^,^8^), the “materials → properties” problem is tackled, and the former design aspect is addressed indirectly through enumeration, i.e., explicit consideration of a large number of candidate materials. Confronting both these hurdles is critical to accelerate, streamline and focus the materials design process.

In the present contribution, strategies are presented to overcome such design challenges for the example of polymer dielectrics—essential components in several applications such as electrical insulation^9^, capacitive energy storage^1^,^10^,^11^,^12^,^13^, organic photovoltaics^14^,^15^, and flexible, stretchable and wearable electronics^16^,^17^. While some polymer dielectrics options are available for these applications, given the vastness of the polymer chemical space, it is extremely likely that significant untapped opportunities remain hidden. A more diverse spectrum (than currently available) of new, better and more suitable candidates will constantly be needed to meet growing future needs mandated by performance measures, amenability to synthesis and compatibility with other parts of devices. Rational and accelerated polymer design strategies and solutions would thus be enormously useful.

Our starting point in the present work is the generation of reference property data (using first principles computations) for a benchmark set of polymers spanning a particular chemical subspace. Interpolative statistical learning concepts^18^,^19^,^20^,^21^,^22^,^23^,^24^,^25^ are then used to train an *on-demand* instant property prediction model using this initial dataset, via an intermediate (and critical) ‘fingerprinting’ step that converts every polymer to a numerical string (c.f. Fig. 1). The prediction scheme produces accurate results for cases not used in the training phase (but falling within the same chemical subspace), as demonstrated by comparisons with more laborious first principles computations and experimental measurements. Such a model via an enumeration scheme can be used to predict the properties of a plethora of new candidate materials in an attempt to search for cases meeting a particular set of property needs. Furthermore, one can make rapid go/no-go decisions on whether a new synthesisable polymer is worth pursuing or not.

The enumeration approach to materials design is not the most efficient one, as it involves consideration of an enormous number of cases, most of which will not be viable in the end (thus leading to low success rates). A better approach is to use the on-demand property prediction scheme within a genetic algorithm^26^,^27^, to directly tackle the “desired properties → suitable materials” design problem. Several polymers that meet a property requirement criterion are designed directly here using such a strategy at a minuscule fraction of the time required for enumeration. The predicted property results of the designed polymers are validated by explicit first principles computations.

The suite of tools and strategies that emerge from this effort take us a step closer to rational, accelerated and direct design of materials in general, and polymer dielectrics in particular. These strategies can also be extended to larger polymer chemical and property subspaces. The essential ingredients of this effort are illustrated in Fig. 1, and described in detail in the following.

## Data Generation

Polymers in our chemical subspace contain a number of linearly repeating chemical building blocks chosen from the following pool: CH_2_, NH, CO, C_6_H_4_, C_4_H_2_S, CS and O. These blocks are commonly found in much of the known polymer space, like polyethylene, polyureas, polythioureas, polyesters, etc. Polymers built from this same pool of blocks were considered by us in the past^1^, but the data from this previous work apply to just individual (i.e., isolated) polymer chains, thus leading to (extrapolated) property data with significant uncertainties. Here, we go beyond the past work, and determine the 3-dimensional packing and crystal structure of polymers arising from these building blocks. Properties computed for such 3-dimensional structures constitute a robust dataset.

For the creation of the initial dataset via first principles computations, we restrict ourselves to 4-block polymers, that is, polymers built with 4 blocks in the repeating unit (with each of these drawn from the pool of 7 building blocks). As described below, the goal of the learning models developed here is to use this dataset to predict the properties of polymers with arbitrarily long repeat units. A total of 406 symmetry-unique 4-block polymers can be formed using the 7 building blocks, of which only 284 were considered here. This reduced number is because chemical intuition and prior knowledge dictates that some combinations of adjoining chemical blocks make for unstable systems, leading to the elimination of all polymers consisting of O-O, CS-CS, CO-CO and NH-NH pairs. The crystal structures of all 284 4-block polymers were determined using the minima hopping method^28^,^29^, with the necessary total potential energies and atomic forces computed using density functional theory (DFT). The structure prediction and DFT details are provided in the Methods section; all the DFT predicted data for the 4-block polymers is provided in the Supplementary Information.

With the 3-dimensional structure of all 284 polymers determined, their relevant properties were calculated. In the present work, we focus on the bandgap (E_gap_), computed using hybrid electron exchange-correlation functionals, and the electronic (*ϵ*_elec_), ionic (*ϵ*_ionic_) and total (*ϵ*_total_ = *ϵ*_elec_ + *ϵ*_ionic_) dielectric constant, computed using density functional perturbation theory, as described in the Methods section. In the case of dielectrics, the bandgap and dielectric constant are the primary properties of interest, generally used in an initial screening stage, regardless of the specific applications^1^,^30^,^31^,^32^.

**Figure 2:**
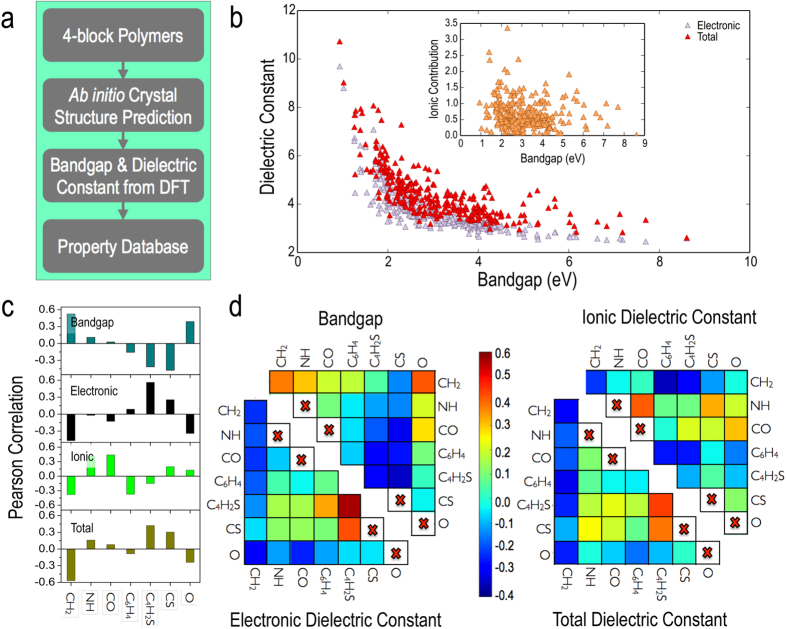
Data generation from DFT and origins of properties. (**a**) The different steps involved in generating a database of the properties of 4-block polymers. (**b**) The DFT computed electronic, ionic and total dielectric constants plotted vs bandgaps for the 4-block polymers. (**c**) Pearson correlation coefficients between fingerprint M_*I*_ and the 4 properties. (**d**) Correlations between fingerprint components of M_*II*_ and the properties shown in the form of heat maps. Red crosses represent the components which lead to unstable polymers and were not considered in the present study (see text for details).

The workflow underlying the data generation step is depicted in Fig. 2a, and the DFT results are portrayed in Fig. 2b. It can be seen from Fig. 2b that *ϵ*_elec_ (shown in purple) seems to follow an inverse relationship to E_gap_, whereas *ϵ*_ionic_ (shown in yellow) has no particular relationship with E_gap_^1^,^32^. Given the larger range of values of *ϵ*_elec_ (2 − 10) than those of *ϵ*_ionic_ (0 − 3), this effect translates to *ϵ*_total_, and we can see an overall inverse relationship between *ϵ*_total_ (shown in red) and E_gap_ as well. For high dielectric constant polymer insulator applications, we are interested in polymers that simultaneously show high *ϵ*_total_ and large E_gap_. Indeed, based on this notion, our past work—although it dealt with isolated polymer chains and estimates of *ϵ*_total_ and E_gap_ in the absence of crystal structure information—has lead to new polymer dielectric solutions^1^,^10^.

## Fingerprinting Polymers

While high-throughput data generation efforts can provide useful ‘lead candidates’ with desired properties, the natural question that arises is whether one can understand the origins of the attractive behavior, and harness this understanding to search for other suitable options. Within the context of polymeric materials under investigation here, the origins should be traceable to the identities of the chemical building blocks. This comes from the theory that electronic and dielectric properties of organic polymers can be effectively expressed in terms of a sum of contributions from different constituent groups^33^. These contributions are in the form of polarisabilities and dipole-dipole interactions from the groups, with different weights attached to different groups. In the case of our polymers, some building blocks, or some combination of blocks, are expected to have a particular influence on the properties being studied.

Thus, if we can numerically represent—or fingerprint—our polymers based on their building block identities, correlations can potentially be established between the fingerprints (or parts of it) and properties. Indeed, numerically representing molecules and materials is emerging as an active topic of inquiry within materials science, physics and chemistry in recent years^34^,^35^,^36^,^37^. Descriptors such as this have historically been used in cheminformatics and related fields like medicinal chemistry and drug discovery^38^,^39^. Key requirements of such representations are that the fingerprints should be intuitive, easily computable, invariant with respect to translations and rotations of the material, invariant to permutations of like atoms or motifs, and generalisable to all cases within the same chemical subspace. Such an approach was recently implemented by us for fingerprinting organic molecules and crystals in terms of constituent atom types (analogous to the chemical building blocks in case of our polymers here)^6^. Singles, doubles and triples of different atom types (based on atom identity and coordination of bonds) were successfully correlated with a number of calculated properties.

A simple polymer fingerprint could therefore be a count of the number of different types of building blocks (e.g., the number of CH_2_ blocks, the number of C_6_H_4_ blocks, etc.), normalised by the total number of blocks in the repeat unit. This would give rise to a 7 dimensional vector, each component of which corresponds to one of the blocks and is related to the number of times it appears in the given polymer repeat unit. We call this fingerprint M_*I*_. While it is a simple and elegant way of representing a polymer, M_*I*_ does not take the effects of neighbouring blocks into account. Thus, we go a step higher in complexity and propose fingerprint M_*II*_, which is a count of the number of different types of pairs of building blocks in the polymer, normalised again by the total number of blocks in the repeat unit. M_*II*_ is defined as a 7 × 7 matrix, every component of which corresponds to any one pair of two neighbouring blocks (eg. CH_2_-NH pairs, CS-O pairs, etc.). Similarly, a fingerprint M_*III*_ can be defined which would be a 7 × 7 × 7 matrix each component of which refers to any triplet of blocks (CH_2_-NH-CO triplets, C_4_H_2_S-C_6_H_4_-CS triplets, etc.).

In this fashion, we could go to higher dimensional fingerprints with more information added at every step; in the limit that we consider *n*-tuple block combinations, we can uniquely represent any polymer out of an *n*-block polymer repeat unit chemical space. We note that this general fingerprinting concept was presented before by us^32^, but only a subset of the fingerprint components (namely, the diagonal) was considered earlier. We argue here that there is no reason to choose such a restricted fingerprint, and moreover, show in the Methods section that the full tensorial representation satisfies key sum rules. With the present prescription, the fingerprint for any given n-block polymer is populated by assigning a certain score to every block or pair of blocks or triplet of blocks that is encountered, with the counting done from either end of the polymer repeat unit to take periodicity and inversion into account. The scores are always averaged and normalised by the total number of blocks in the repeat unit. The averaging step ensures that sum rules are satisfied, and normalisation assures that the fingerprints are generalisable to repeat units of arbitrary length. It should be noted that this polymer fingerprint does not take into account spatial degrees of freedom or other structural factors, and would thus not distinguish between two polymers with the same repeat unit but different crystal structural arrangements.

For ease of initial discussion, we consider the fingerprints M_*I*_ and M_*II*_. Correlations between the different components of fingerprint M_*I*_ and 4 properties (*ϵ*_elec_, *ϵ*_ionic_, *ϵ*_total_ and E_gap_) are shown in Fig. 2c. The coefficients plotted on the *y*-axes were obtained using the Pearson correlation analysis, which gives us values between −1 and +1 showing the degree of negative or positive correlation between any property and any component of the fingerprint vector. The opposite behaviour of *ϵ*_elec_ and E_gap_ can be ascertained by observing their respective plots: CH_2_ and O blocks make notable positive contributions to E_gap_ and negative contributions to *ϵ*_elec_, whereas C_4_H_2_S and CS contribute positively to *ϵ*_elec_ and negatively to E_gap_. The same effects largely translate to *ϵ*_total_ as well while for *ϵ*_ionic_, CO and NH blocks contribute the most.

Results for a similar Pearson correlation analysis between M_*II*_ and the 4 properties are shown in Fig. 2d in the form of half-matrix heat maps. The shade of the colour in any matrix component (based on the adjoining colour scale) shows how positively or negatively that particular pair of blocks is correlated with the given property. Once again, it can be seen how the heat map for E_gap_ is really opposite to that of *ϵ*_elec_ or *ϵ*_total_ in terms of the spectrum of colours (dark blue to dark red). While C_6_H_4_-C_4_H_2_S, C_4_H_2_S-C_4_H_2_S and C_4_H_2_S-CS pairs make the most positive contributions to *ϵ*_elec_ and CH_2_-O and CO-O pairs make the most negative contributions, the roles of these pairs are just reversed when considering their contributions to E_gap_. In case of *ϵ*_ionic_, NH-CO, NH-CS and CO-O pairs contribute to its increase while CH_2_-C_6_H_4_ and CH_2_-C_4_H_2_S pairs have the opposite effect. It is now possible for us to come up with educated combinations of different kinds of pairs of building blocks targeted towards increasing the dielectric constant or the bandgap or indeed, both. In light of these insights, it is not surprising that polymers with [-NH-CO-NH-C_6_H_4_-], [-NH-CS-NH-C_6_H_4_-], and [-NH-CO-NH-C_6_H_4_-] repeat units were singled out in past work as promising dielectrics for energy storage applications^1^.

## On-Demand Property Prediction

While qualitative notions such as discussed above are useful, a quantitative property prediction model that is fast (because it by-passes the DFT route to property predictions) would satisfy several practical needs. Following previous work, we use kernel ridge regression (KRR)^40^ to establish a quantitative mapping between the polymer fingerprints on the one hand and the relevant properties (namely, E_gap_, *ϵ*_elec_, and *ϵ*_ionic_) on the other. KRR is a statistical or machine learning algorithm capable of handling non-linear relationships^6^,^32^. By comparing the fingerprint, say *M*_*III*_, of a new polymer with those of a set of reference cases for which property values are known, an interpolative prediction of the property of the new polymer may be obtained. In practice, the machine learning prediction model is developed for a subset of the available dataset, referred to as the training set, and the performance of the model is tested on the remainder of the dataset, referred to as the test set. Model development based on the training set also included internal cross-validation to minimise over-fitting and ensure model generality. In the present work, about 90% of the 284 4-block polymer dataset was taken to be the training set, and the remaining 10% was placed in the test set. The optimal training set size was determined by studying the ML model performances for different training set sizes; this data is presented in the Supplementary Information. Further details of the KRR method and specifics of the model development are provided in the Methods section.

**Figure 3:**
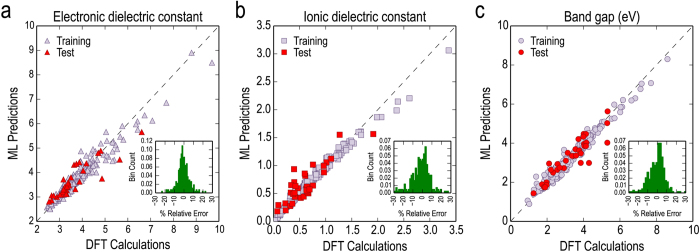
Prediction model performances. Comparison of the KRR property predictions with DFT evaluated properties for the prediction models for *ϵ*_*elec*_, *ϵ*_*ionic*_ and E_*gap*_ respectively.

The plots in Fig. 3 show E_gap_, *ϵ*_elec_ and *ϵ*_ionic_ as predicted using the KRR-based machine learning (ML) model (and fingerprint M_*III*_) versus the respective DFT values. The insets also show the relative error distribution for each property prediction, indicating that the average error for all three properties is of the order of 10% or less. We thus have a model in our hands that will convert a fingerprint (M_*III*_, in the present illustration) to property values with errors that are reasonable (given the efficiency of the prediction process relative to DFT). Prediction performances using M_*I*_ and M_*II*_ for KRR are shown in the Supplementary Information for completeness.

**Figure 4:**
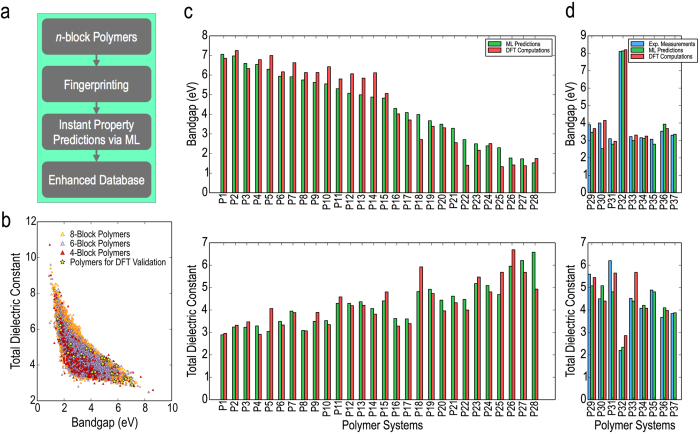
On-demand property prediction of polymers. (**a**) The steps involved in predicting properties of any given *n*-block polymer using the instant prediction models. (**b**) Dielectric constants and bandgaps from the prediction models plotted against each other for all 6-block polymers and 8-block polymers, with the computational data for 4-block polymers also shown for reference. (**c**) Machine learning predicted and DFT computed properties of 28 polymers obtained by applying the direct design scheme to different ranges of dielectric constants and bandgaps. (**d**) The machine learning predicted, DFT computed and experimentally measured properties of some previously synthesised polymers.

The true power of such a property prediction model is its ability to instantly predict E_gap_, *ϵ*_elec_ and *ϵ*_ionic_ for a polymer with arbitrarily long repeat unit (but with the building blocks drawn from the same pool of 7), without needing to pursue the cumbersome approach of structure prediction and DFT. The workflow involved in predicting the properties of new *n*-block polymers is depicted in Fig. 4a. If one were to pursue the enumeration approach, it is straightforward to list all possible *n*-block polymers for any given *n*, as long as *n* is a small enough number. To illustrate this, we came up with all the possible symmetry-unique 6-block polymers (~6000 in number) and 8-block polymers (~150000 in number), determined their respective fingerprints, and estimated their properties using our ML model. Figure 4b shows the predicted *ϵ*_total_ (=*ϵ*_elec_ + *ϵ*_ionic_) plotted against the predicted E_gap_ values for all the 6-block polymers and 8-block polymers, as well as for the considerably smaller number of the 4-block polymers. Figure 4b is a demonstration of how one may use interpolative statistical learning methods to densify the population within a chemical subspace. We thus have a lot more options to choose from.

The predictive performance of our model can be put to test in two ways: by comparing our predictions with actual DFT calculations, and by comparing them with available laboratory measurements. First, we validate our ML model against DFT calculations. A selection of 8-block polymers ranging from low (high) to high (low) values of *ϵ*_total_ (E_gap_) was chosen out of Fig. 4b (shown by stars in figure; incidentally, these were also the cases identified by our genetic algorithm, discussed in the next section, but the same examples serve the present purpose of ML model validation). The stable crystal structures of these 8-block polymers were determined, following which their properties were calculated using DFT. Figure 4c compares the ML prediction with the corresponding DFT results. *As can be seen, the agreement is impressive indicating that the prediction model trained on 4-block cases is transferable to polymers with repeat units of arbitrary size.*

Next, in Fig. 4d, we compare the on-demand predictions with experimental values for polymers synthesised and tested in the recent past^1^,^10^, as well as the corresponding DFT results, for completeness. These polymers were synthesised following the earlier work^1^ on high-throughput computational data generation using the isolated polymer chains model; this means we have available experimental as well as computational quantification of *ϵ*_total_ and E_gap_ for a number of polymers which are predictable with our prediction models. Clearly, again, the performance of the ML model is impressive. The closeness of our predictions with first principles as well as with actual experiments allows us to state with some confidence that we have the means to instantly, and with reasonable accuracy, predict the properties of any *n*-block polymer belonging to the chemical subspace under consideration. All the polymers plotted in Fig. 4c,d are denoted by some labels, and the polymer corresponding to each label is mentioned in Table 1. The ML predictions are always close to the experimental values, validating our claim of accelerating property prediction for arbitrarily long polymer chains.

## On-Demand Direct Design

Although the entire expanse of the chemical space can be covered using enumeration, it is essentially a brute-force search for suitable polymers, and as such not the best possible design strategy. For instance, enumerating for 8-block, 10-block and 12-block polymers will lead to ~1.5 × 10^5^, 5 × 10^6^ and 5 × 10^7^ systems respectively, which are unreasonably large numbers considering the property domain of interest may restrict us to a small fraction of that. We thus attempted to find an efficient way of obtaining specific *n*-block polymers that simultaneously show a certain desirable dielectric constant and a desirable bandgap, without having to individually consider every possible polymer. Such a model would make the “desired properties → suitable materials” route an instant, on-demand reality^5^,^6^,^7^,^8^.

**Figure 5:**
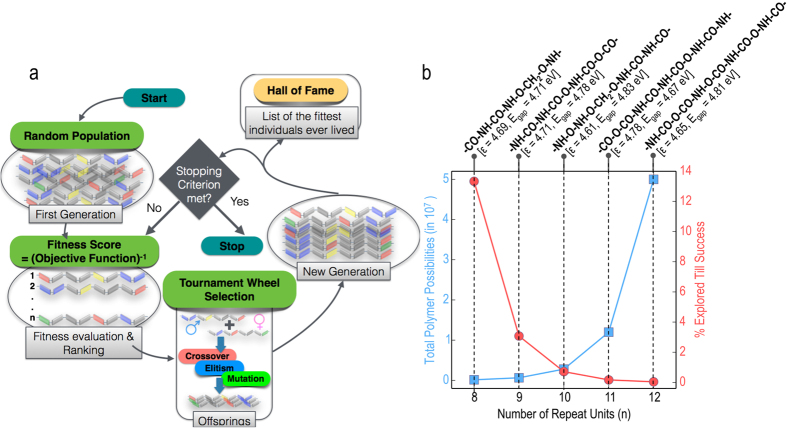
On-demand direct design of polymers. (**a**) The steps involved in the genetic algorithm (GA) approach leading to direct design of polymers. (**b**) The exponential increase in total polymer possibilities for increasing number of repeating blocks, and the simultaneous decrease in the percentage of points to be explored till success. Also shown are one optimal polymer each for each case for a target dielectric constant and bandgap of 5 and 5 eV respectively.

We applied a genetic algorithm (GA) approach as the means to optimise the polymers given the target properties. It has been shown that GA is a very efficient approach in searching for materials with desired properties when compared to other approaches like random search and even chemical-rules based search^26^. The idea here is to start with a random initial population of *n*-block polymers (for any given *n*) and let them undergo evolution (in terms of constituent blocks and their neighbours) based on the principles of GA, finally yielding a set of polymers with properties closest to the provided targets. At any step, the properties of the polymers are computed instantly using the on-demand prediction ML model we developed and explained in the previous section. The series of steps followed in this method are shown in Fig. 5a. In an earlier work^6^, we implemented the same philosophy but used a simulated annealing approach instead of GA for designing organic molecules with specific target properties.

Given the target *ϵ*_total_ and E_gap_, and the number of blocks in the polymer repeat unit (the value of *n*), the algorithm generates a list of 300 *n*-block polymers which serves as the first generation. Based on the predicted property values, a fitness score (explained in detail in Methods) is assigned to every polymer and all the polymers are ranked according to this score. While polymers with satisfactory fitness scores survive (this is called elitism), the rest undergo different kinds of evolution, namely crossover and mutation (again, explained in Methods). New generations of polymers are produced in this manner; a stopping criterion is provided based on the fitness score, and once polymers with suitable fitness scores are obtained, the algorithm stops. From every generation, the polymers with fitness scores above a certain threshold are compiled as the list of best solutions. At the end of the algorithm, this list contains the final set of optimal polymers showing the desired *ϵ*_total_ and E_gap_.

For a demonstration and validation of this approach, we restricted our initial search to 8-block polymers only, as this provides us with a substantial population of systems to explore while ensuring the system size does not become so large as to render subsequent first principles validation extremely expensive. We took 6 different (*ϵ*_total_, E_gap_) combinations as the targets, and allowed the algorithm to search for suitable 8-block polymers showing each combination of properties. Figure 4c gives a glimpse of the results: we show a few polymers each obtained for the different targets we provided. The ML model predicted property values for these polymers are always close but not exactly the same as the target values—but these are the polymers showing the highest fitness scores for the given targets. A comparison between the solutions obtained from the genetic algorithm approach and the solutions obtained from the enumerated list of all 8-block polymers, is shown in the Supplementary Information. It is seen that for a given target property set, this scheme does indeed determine a number of optimal solutions, if not all of them.

To understand exactly how valuable the direct design scheme is, we need to quantify the speed of the GA approach when compared to enumeration. Taking the example of 8-block polymers, while there are a total possible ~150000 such systems, GA is able to traverse a small percentage of the points in determining the required polymer(s). Upon going to higher block systems, like 9-block or 10-block polymers, the total possibilities are exponentially higher but the percentage of points the algorithm needs to explore is even smaller. Figure 5b shows that despite the exponential increase in total polymer possibilities, as the number of repeating units *n* increases, a smaller and smaller percentage of points need to be considered by the algorithm in order to obtain the optimal polymer(s). Also shown in Fig. 5b are *n*-block polymers obtained for different values of *n* for a target *ϵ*_total_ of 5 and a target E_gap_ of 5 eV. Thus, with actual polymer outputs (with arbitrarily long chains) as well as a quantification of the speed-up, we have in our hands an efficient polymer design model that negates the need for enumeration followed by down-selection of desired systems.

## Summary

Given a material, we want to have the means to instantly estimate its properties, and thus make a quick decision on its suitability for an application. We have demonstrated how carefully created and curated materials data can be used to train statistical learning models. These models, following testing and validation, require merely the fingerprint of a new material to output its properties. We have further shown how a genetic algorithm can be combined with the learning models to determine specific materials that possess a certain set of desired properties. In this manner, using the example of polymer dielectrics, we have successfully tackled both the “desired properties → suitable materials” and the “materials → properties” problems. The materials design philosophy applied in this work can be used for any class of materials, as long as there is sufficient data available for training, and an intuitive and easily attainable material fingerprint can be proposed.

We have at our disposal a polymer database, generated from first principles and expanded substantially using the on-demand prediction model, which enables us to recommend a number of new polymers previously not considered for synthesis and testing. While this fulfills one of the specific aims we set out to achieve, that is expand on the list of promising dielectric polymers, the prediction and design models can prove to be very valuable tools for polymer synthesists. Not only can they make an instant go/no-go decision on any polymer, but they can actively seek specific polymers that would suit their requirements. This adds a new, very useful dimension to the field of dielectric polymer design.

There are of course limitations to the models, not least in terms of the chemical blocks currently considered. The information in the models, and thus the possible guidance, is restricted to combinations of only the 7 basic building blocks. For an extension to more blocks, sufficient amount of data on polymers containing those blocks needs to be generated, and the models retrained. Further, the fingerprints currently used only take into account the constituent blocks in the polymers, and exclude information on how the polymer chains are stacked against each other, and other factors that may affect the properties. While this is indeed a limitation, we argue that such a fingerprint still functions very well, and points to the fact that materials can typically be boiled down to fairly simple numerical representations. Lastly, it must be noted that all property predictions from the on-demand prediction model come with some uncertainties, which are inevitable in any statistical learning enterprise. Nevertheless, we have established a useful materials design protocol that should help accelerate the design and discovery of polymers encompassing a much larger chemical subspace, or non-polymeric but quasi-1D systems (e.g., superlattice heterostructures).

## Methods

### First principles computations

A unit cell is set up containing 2 polymer chains stacked next to each other. The Minima Hopping structure prediction algorithm^28^,^29^,^41^,^42^ was applied on the starting polymer geometry, leading to the exploration of many low energy crystal structure arrangements which were ranked according to their relative energies. For each polymer, the lowest energy crystal structure thus obtained was taken for DFT property calculations. DFT^43^ as implemented in the Vienna ab initio software package (VASP)^44^ was applied, and relaxation was performed using the rPW86 functional wherein the DFT-DF2 vdW correction is applied^45^ to capture the van der Waals interactions in the polymer correctly^46^. We used projector-augmented wave (PAW)^47^ pseudopotentials and imposed a tight energy convergence criterion of 10^−8^ eV and an energy cut-off of 500 eV. The relaxed geometry thus obtained went as input into a subsequent density functional perturbation theory (DFPT)^48^ calculation, which provided us with the dielectric constant tensor that includes the electronic component^49^ as well as the ionic (lattice) component^50^. The reported dielectric constant values were obtained by determining the trace of the respective dielectric tensor (a 3 × 3 matrix in this case). Further, the Heyd-Scuseria-Ernzerhof (HSE)^51^ functional was used on the relaxed geometries to obtain the HSE bandgap values, which are known to be more reliable^52^.

### Fingerprint

M_*I*_, M_*II*_ and M_*III*_ are characterised by a number of key mathematical constraints which have been listed below-

1. The sum of all the elements in any fingerprint should be equal to the total number of blocks in the polymer (N). Thus: 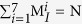, 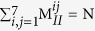 and 
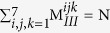
.
2. The sum of elements in any row or column of M_*II*_ should be equal to the total number of blocks of that kind in the polymer. This can be written as: 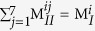. Similarly, the sum of elements in any given 7 × 7 matrix plane in M_*III*_ should be equal to the total number of blocks of that kind in the polymer, which can be written as: 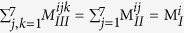.
3. The periodic symmetry in the polymer dictates that the fingerprint matrix diagonal acts as a mirror; the corresponding elements on either side of it should be equal. That is, 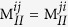 and 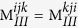.
4. The diagonal elements in any fingerprint matrix should be integer values, that is, 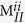 and 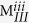 ∈ the set of non-negative integers.

### Regression

Kernel Ridge Regression (KRR) was applied to develop a similarity-based model, where the Euclidean distances between fingerprints are used to compute a distance Kernel. In this work, a Gaussian Kernel was used. A given property is then expressed as a weighted sum of the Gaussians. The different parameters that go into the training of such a model— the Gaussian width parameter, the regularisation parameter (which helps to prevent overfitting in the data)^40^, and the coefficients of the Gaussians— are changed in a systematic manner so as to achieve maximum closeness between the weighted sum of the kernels and the property. From our database of 284 4-block polymers, all the points were randomly divided into two sets— the training set (250 points) and the test set (34 points). The training set was used to train the KRR model and thus come up with the prediction model with the minimum error in property prediction. The best models thus obtained were used to predict the properties on a test set in order to evaluate their true out-of-sample performance. To ensure the best possible training in an unbiased manner, a cross-validation technique was used where the training set itself is divided into two sets and one set is used for preliminary training with validation done on the other.

### Genetic Algorithm

Based on the target dielectric constant and bandgap, an objective function was defined as the following-


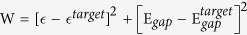


where *ϵ*^target^ and 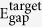 are the target dielectric constant and bandgap values respectively, while *ϵ* and E_gap_ are the dielectric constant and bandgap of the polymer undergoing optimisation. This function would be minimised when the difference between either property of the polymer and the respective target property is the least. Further, a *Fitness Score* was defined as the inverse of the objective functional value, and acted as the measure of suitability of any system. We devised a polymer encoding system that converted any *n*-block polymer into an *n*-component vector, assigning a number between 0 and 6 to each of the 7 motifs respectively. Using completely random values for this vector, an initial population of 300 polymers was generated. Properties were instantly calculated for all these polymers using the on-demand prediction models, and the fittest polymers (showing the highest fitness scores) were selected. Mating is performed between these individuals using a combination of crossovers, elitism and mutation^26^, giving rise to the ‘offspring’ polymers that then go forth to the next generation of polymers. In crossover, some of the vector components of the parent polymers were simply exchanged to generate the children. Elitism means preserving a few of the fittest parent polymers in the next iteration, whereas with mutation, we changed some of the vector components of the parents randomly to obtain the children. Thus, generation after generation of polymers was studied and those with the highest fitness scores at every generation went into the list of best solutions. In the end, this list would contain the best individuals that ever lived (that is, the polymers with properties closest to the target values *ϵ*^*target*^ and 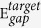), and these would be our solutions.

## Additional Information

**How to cite this article**: Mannodi-Kanakkithodi, A. *et al.* Machine Learning Strategy for Accelerated Design of Polymer Dielectrics. *Sci. Rep.* **6**, 20952; doi: 10.1038/srep20952 (2016).

## Supplementary Material

Supplementary Information

Supplementary Datset 1

## Figures and Tables

**Figure 1:**
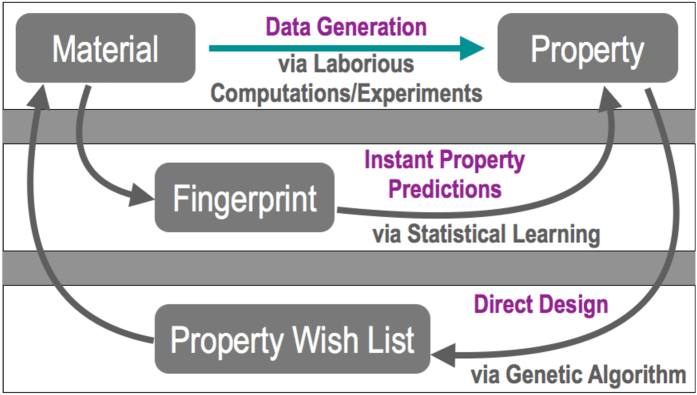
The overall outline of this work. This work is divided into three stages: the data generation stage, the instant property prediction stage and the direct design stage.

**Table 1:** Polymer repeat units denoted by the labels P1 to P37 in Fig. 4c,d.

Label	Polymer Repeat Unit	Label	Polymer Repeat Unit
P1	CH_2_-O-CH_2_-O-CH_2_-CH_2_-CH_2_-CH_2_	P20	O-C_6_H_4_-CO-C_4_H_2_S-CO-NH-O-CO
P2	CH_2_-O-CH_2_-O-CH_2_-CH_2_-CH_2_-O	P21	CH_2_-CH_2_-O-CS-NH-CS-C_6_H_4_-NH
P3	CH_2_-NH-CH_2_-CH_2_-CH_2_-O-CH_2_-O	P22	C_6_H_4_-C_6_H_4_-CH_2_-CS-C_4_H_2_S-CS-CH_2_-O
P4	CH_2_-CH_2_-O-CO-O-CH_2_-CH_2_-O	P23	C_6_H_4_-NH-C_6_H_4_-CS-NH-C_4_H_2_S-CO-NH
P5	CO-O-CH_2_-CH_2_-CH_2_-CH_2_-CH_2_-O	P24	CO-C_4_H_2_S-NH-CS-O-C_4_H_2_S-NH-C_4_H_2_S
P6	CH_2_-CH_2_-O-CO-NH-CH_2_-CH_2_-O	P25	CS-CO-CH_2_-CH_2_-NH-C_6_H_4_-CS-C_6_H_4_
P7	CH_2_-NH-CO-NH-CH_2_-O-CH_2_-O	P26	C_6_H_4_-NH-C_4_H_2_S-C_4_H_2_S-CS-C_4_H_2_S-C_4_H_2_S-NH
P8	CH_2_-CH_2_-CH_2_-CH_2_-NH-CO-CH_2_-CH_2_	P27	C_4_H_2_S-C_4_H_2_S-C_4_H_2_S-CS-C_4_H_2_S-NH-CS-NH
P9	CO-NH-O-CH_2_-CH_2_-CH_2_-CH_2_-O	P28	C_4_H_2_S-CS-C_4_H_2_S-CS-CO-NH-C_6_H_4_-C_4_H_2_S
P10	CH_2_-O-CO-NH-CH_2_-CH_2_-NH-CH_2_	P29	NH-CO-NH-C_6_H_4_
P11	CH_2_-NH-CH_2_-NH-CO-NH-CO-NH	P30	CO-NH-CO-C_6_H_4_
P12	CH_2_-NH-CO-O-NH-CO-NH-O	P31	NH-CS-NH-C_6_H_4_
P13	CO-NH-CO-O-CO-NH-CH_2_-NH	P32	CH_2_-CH_2_-CH_2_-CH_2_
P14	CO-NH-CO-NH-CH_2_-CH_2_-CH_2_-NH	P33	NH-CS-NH-C_6_H_4_-NH-CS-NH-C_6_H_4_-O-C_6_H_4_
P15	CO-NH-CO-CH_2_-NH-CO-O-NH	P34	NH-CS-NH-C_6_H_4_-NH-CS-NH-C_6_H_4_-CH2-C_6_H_4_
P16	C_6_H_4_-O-CO-CH_2_-CO-CH_2_-CH_2_-O	P35	NH-CS-NH-C_6_H_4_-NH-CS-NH-C_6_H_4_
P17	CH_2_-CH_2_-CO-O-CO-CH_2_-C_6_H_4_-C_6_H_4_	P36	NH-CS-NH-C_6_H_4_-NH-CS-NH-[CH_2_]_6_
P18	CO-NH-O-NH-CO-NH-C_4_H_2_S-NH	P37	NH-CS-NH-C_6_H_4_-CH_2_-C_6_H_4_
P19	CO-NH-CO-NH-CO-NH-C_4_H_2_S-NH		
