# Supplementary material for: Machine Learning Strategy for Accelerated Design of Polymer Dielectrics
Source: Sci Rep. 2016 Feb 15;6:20952. doi: 10.1038/srep20952 (PMC4753456; doi:10.1038/srep20952)
Supplement: Supplementary Information [file srep20952-s1.doc]

**SUPPLEMENTARY INFORMATION**

**Title of Manuscript**

Machine Learning Strategy for Accelerated Design of Polymer Dielectrics

**Authors**

Arun Mannodi-Kanakkithodi^1^, Ghanshyam Pilania^2^, Tran Doan Huan^1^, Turab Lookman^3^ & Rampi Ramprasad^1^

^1^Department of Materials Science and Engineering, Institute of Materials Science, University of

Connecticut, 97 North Eagleville Road, Storrs, Connecticut 06269, USA

^2^Materials Science and Technology Division, Los Alamos National Laboratory, Los Alamos, New Mexico 87545, USA

^3^Theoretical Division, Los Alamos National Laboratory, Los Alamos, New Mexico 87545, USA

1. **Supplementary Data** (all the DFT data on the 4-block polymers)

Structure prediction was performed for all the 284 4-block polymers. On the most stable crystal structure obtained for each polymer, DFT was used to estimate the dielectric constants (divided into the electronic part, ε_elec_ and the ionic part, ε_ionic_, with the total dielectric constant ε_total_ = ε_elec_ + ε_ionic_) and the bandgap values (in eV). The sheet labeled ‘All DFT Data’ in the excel file provided along with this supplementary document lists the chemical repeating unit and the four properties for all the polymers.

1. **Supplementary Discussion**
   1. **Optimal Fingerprint Selection**

The best KRR prediction performances that were obtained after testing (and thus used in developing the final prediction and design models) were using fingerprint M_III_ and a training set size of 250 points. For completion, we show in Fig. 1 and Fig. 2 the respective KRR prediction performances using fingerprints M_I_ and M_II_ for each of the three properties, with a training set size of 250 point again. Although the performances are fairly reasonable, the prediction errors are slightly higher than with M_III_, and using M_III_ as the optimal fingerprint is justified.


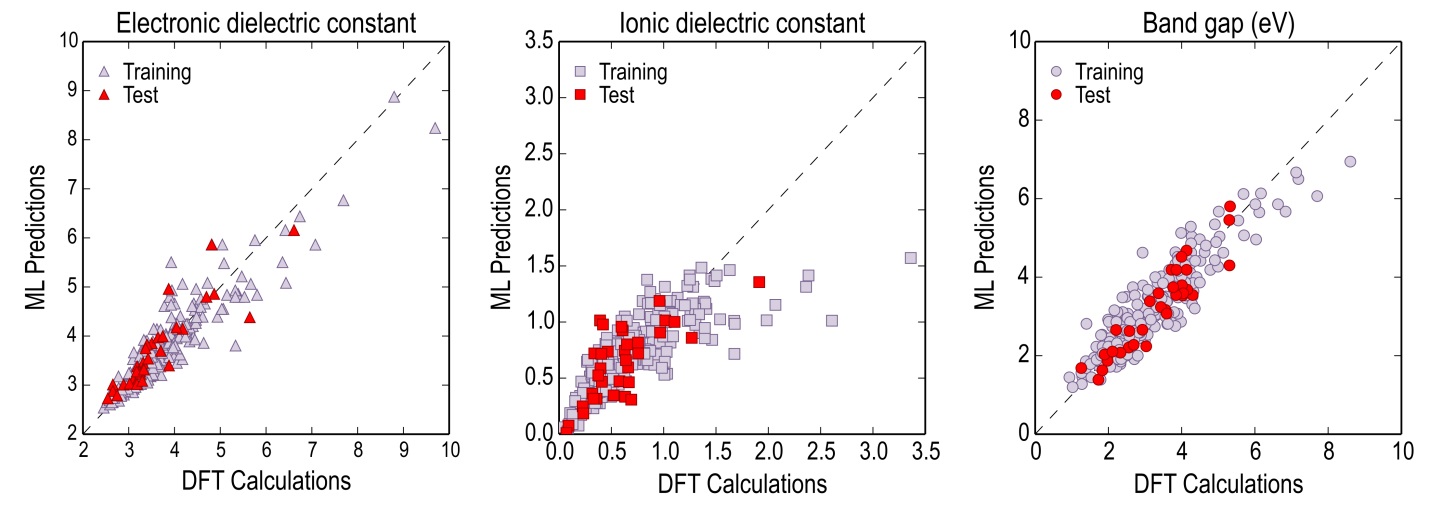


Fig. 1: Comparison of KRR predictions with DFT evaluations for model trained with M_I_.


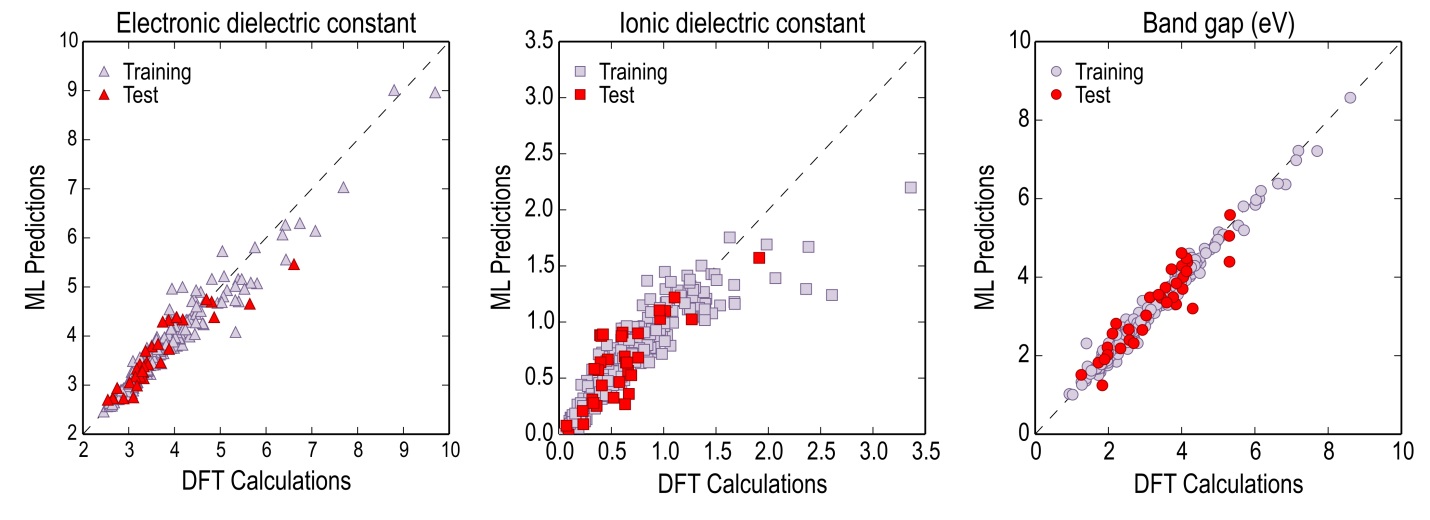


Fig. 2: Comparison of KRR predictions with DFT evaluations for model trained with M_II_.

- 1. **Optimal Training Set Size Selection**

Using the optimal fingerprint M_III_, we varied the size of the dataset used for training the KRR model from 100 to 280 data points, and computed the mean absolute errors on predictions made for the test dataset. In the sheet labeled ‘Learning Curves’ in the excel file provided along with this supplementary document, we list these test-set prediction errors along with the training-set cross validation errors for each of the three properties (i.e., the bandgap and the electronic and ionic parts of dielectric constant). It can be seen that the test errors progressively go down as the training set size is increased, and appear to achieve saturation around the 250 points mark. Thus, an optimal training set size of 250 was chosen for KRR.

- 1. **Global Optima Check for Genetic Algorithm Approach**

In the sheet titled ‘GA Optima Validation’ in the excel file provided along with this supplementary document, we list the 15 polymers obtained using the genetic algorithm (GA) approach for a target dielectric constant of 6.5 and a target band gap of 1.5 eV. Also listed separately, in red, are 8 other polymers (selected out of the list of ~200,000 8-block polymers that were enumerated) that represent ‘equally good minima’ but were not determined by GA. Since the initial population of polymers is generated randomly, the algorithm may not determine all the global minima at the end, but rather a subset of it. Regardless, we believe our algorithm works very efficiently in finding the desired optimal solutions and is thus of great utility in designing polymers.
